# A Comparative Evaluation of the Fracture Resistance of Mineral Trioxide Aggregate (MTA) Plus and MTA Angelus: An In Vitro Study

**DOI:** 10.7759/cureus.40385

**Published:** 2023-06-13

**Authors:** Saurabh Joshi, Dhananjay Gandage, Esha Thakare, Preetam A Mahagaonkar, Rohit Gadda, Aparna U Palekar

**Affiliations:** 1 Department of Pediatric Dentistry, Rural Dental College, Pravara Institute of Medical Sciences, Loni, IND; 2 Department of Prosthodontics, Dr D. Y. Patil Dental College & Hospital, Dr. D. Y. Patil Vidyapeeth, Pimpri, IND; 3 Department of Oral Pathology and Microbiology, SGP Nanded Rural Dental College and Research Centre, Nanded, IND; 4 Department of Prosthodontics, SMBT Dental College and Hospital, Sangamner, IND; 5 Department of Oral Medicine and Radiology, Mahatma Gandhi Mission Dental College and Hospital, Navi Mumbai, IND; 6 Department of Conservative Dentistry and Endodontics, Rural Dental College, Pravara Institute of Medical Sciences, Loni, IND

**Keywords:** immature teeth, endodontics, fracture resistance, mta angelus, mta plus

## Abstract

Background

Mineral trioxide aggregate (MTA) is a biocompatible dental material used for root-end filling in endodontics. A wide variety of literature has been published on the assessment of fracture resistance of MTA. However, the results were conflicting in the reported studies, and the sample size used was insufficient to conclude the efficacy of materials such as MTA Plus and MTA Angelus. Therefore, this study was designed to compare and evaluate the effectiveness of two commercially available MTAs, namely, MTA Plus (Avalon Biomed Inc. by Prevest Denpro Ltd, Jammu, India) and MTA Angelus (Angelus Dental Solutions, Brazil) in terms of fracture resistance.

Methodology

To determine fracture resistance, 300 freshly extracted healthy human teeth with single roots and canals were collected by simple random sampling. Teeth were decoronated, the apical third was enlarged, and root canals were prepared to receive MTA as a 5 mm apical filling. The root segments were randomly categorized into two experimental groups of 100 samples each, namely, group A (MTA Plus) and group B (MTA Angelus), and the remaining 100 root segments were used as control (unfilled). Fracture resistance was determined using the Instron Universal testing machine.

Results

The results of our study showed statistically significant increased fracture resistance for MTA Plus (532.14 ± 5.19 N) than MTA Angelus (540.81 ± 3.56 N) and the control group (460.63 ± 7.91 N).

Conclusions

The control group showed the least fracture resistance. The composition and structure of MTA Angelus (group B) containing Portland cement, with a 4:1 addition of bismuth oxide, make it more fracture resistant than MTA Plus (group A).

## Introduction

Treating teeth with immature root apex and lack of pulp endodontically is challenging for clinicians. Calcium hydroxide was used as a conventional apexification agent. However, the main problems with calcium hydroxide are the prolonged treatment time, leaky restoration, difficulty in protecting thin roots from fracture, and non-homogenous apical seal, leading to failure [1]. Mineral trioxide aggregate (MTA) was initially used and promoted in endodontics due to its efficacy in various procedures, including apexification. A single-visit apexification procedure with MTA can be an alternative to the traditional endodontic practices of calcium hydroxide. However, difficulties exist in treating underdeveloped open apices, with immature roots more prone to fracture due to thin radicular dentinal walls. MTA improves fracture resistance when placed as an apical barrier [1,2]. Matt et al. showed that the fracture strength of the teeth increased after one year when MTA was used as an apical barrier [3]. In addition, MTA induces the expression of tissue inhibitor of metalloproteinase 2 in the dentin matrix, possibly preventing fracture [3]. Different formulations of MTA have given different results; therefore, we compared the fracture resistance of MTA Plus (Avalon Biomed Inc. by Prevest Denpro Ltd., Jammu, India) and MTA Angelus (Angelus Dental Solutions, Brazil) in our study. The difference between MTA Plus and MTA Angelus was in the composition of the two, the setting time of the materials, and the fracture resistance of the materials. Because MTA Plus and MTA Angelus are readily available and economical and few studies have reported on this topic, this study was designed to compare the fracture resistance of MTA Plus and MTA Angelus.

## Materials and methods

The study was conducted in the Department of Pediatric Dentistry, Rural Dental College, in collaboration with the Department of Microbiology, Rural Medical College, Loni, and Indian Institute of Technology (IIT) Powai, Mumbai, after obtaining clearance from the Institutional Ethical Committee, PIMS (Deemed to be University), Loni (PMT/PIMS/IEC/2017/440) and after the confirmation of registration in PhD course in Rural Dental College, PIMS (DU) (PIMS/Ph.D/R/2018/260). A trained dental practitioner along with the laboratory technician were the service providers. Both service providers had undergone training for the same.

A total of 300 freshly extracted single-rooted and single-canal sound human teeth were collected from the Department of Oral and Maxillofacial Surgery. The sample size was calculated by performing a power analysis. Assumptions of sample size calculation were effect size of 0.25, α error of 5%, and power of 80%. G Power 3.1.9.7 calculator was used for sample size calculation. The sampling was done by a simple random sampling method. Immediately after the extraction of the teeth, the teeth were collected, washed, and stored in sodium hypochlorite (5%) for the initial two hours. The storage of the teeth did not affect the outcome of the treatment. The teeth were cleaned by ultrasonic scaling and autoclaved to remove soft tissue tags, calculus, and attached bone or debris. Then, the teeth were stored in 0.1% thymol (VDH Industries Ltd., India) for one month, followed by normal saline (Nirlife, Nirma Ltd., India) until further use. Non-carious extracted teeth, teeth with complete root formation, single root, and canal extracted due to orthodontic reasons (with prior consent for extraction from the patient) were included in the study. Fractured teeth with cracked roots were excluded from the study.

Methodology and grouping

**Figure 1:**
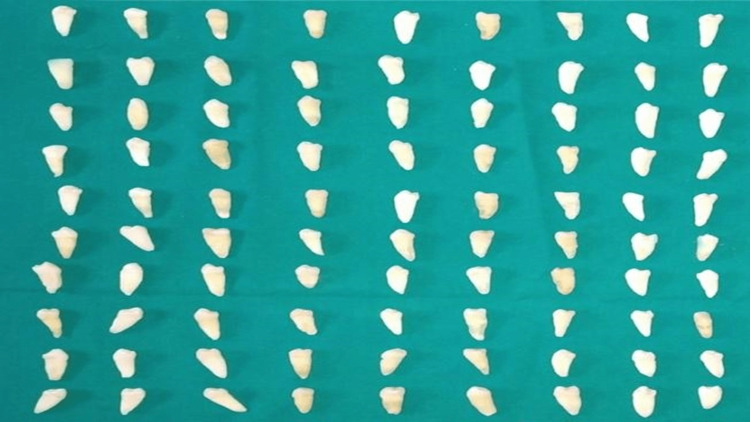
Single-rooted, single-canal teeth.

Decoronation of teeth to 10 mm root length with a Micro mega (MM) handpiece and the diamond disc was performed, followed by a simulation of the root segments with Gates glidden (GG) burs 5-1 in a crown-down manner to the clinical situation of open apices. #1 GG bur was used to pass through the apical foramen. The root segments were prepared with Flexofiles until an ISO size 90 file (Mani Inc, Japan) was visible 1 mm past the apex. Sodium hypochlorite 5% was used as an irrigant throughout the procedure. All root canals were prepared with a step-down technique using Protaper gold (sequence S1, S2, F1, taper 0.06). The specimens received a final rinse with 1 ml 17% ethylenediaminetetraacetic acid solution to remove the smear layer, followed by normal saline (Figure 1).

The 300 root segments for fracture resistance were randomly assigned into two experimental groups (group A and group B) with 100 samples in each group, and 100 samples were used as a control.

**Figure 2:**
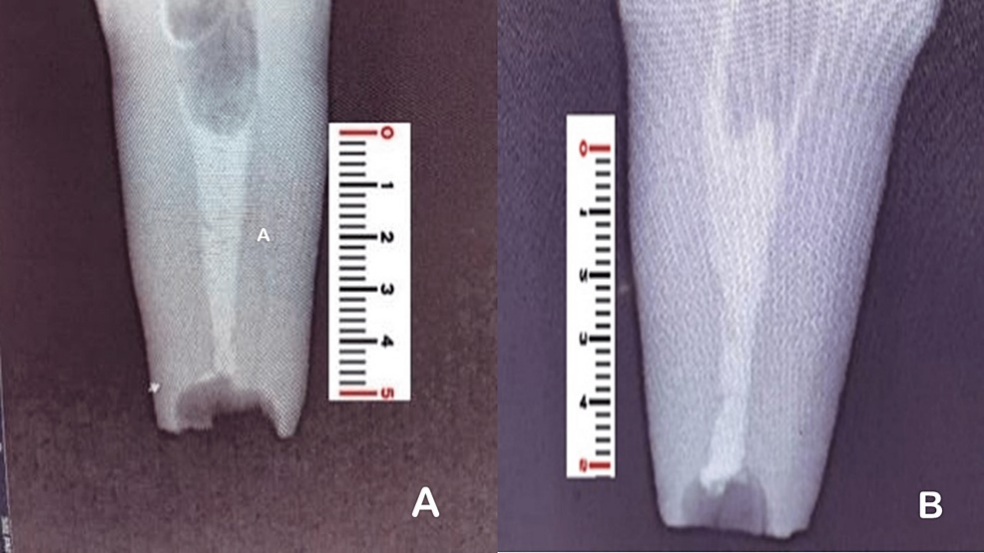
(A) MTA Plus condensed at the apical third. (B) MTA Angelus condensed at the apical third. MTA = mineral trioxide aggregate

In group A, MTA Plus was placed as a 5 mm apical barrier in 100 root segments. In group B, MTA Angelus was placed as a 5 mm apical barrier in 100 root segments. In group C, no material was placed as an apical barrier in 100 root segments, and the obturation was carried out by a lateral condensation method. All materials were manipulated as per the manufacturer’s instructions. The MTA used in both groups was mixed on a paper pad with distilled water in a 3:1 powder-water ratio. When the mixture exhibited putty-like consistency, it was immediately placed as an apical barrier. MTA was placed in the site with the bone graft carrier. The material was condensed gently at the simulated open apex by a plugger with a wet cotton pellet. An apical plug of at least 5 mm was formed. Radiographs were obtained to verify the placement of the apical barriers (Figures 2A, 2B).

**Figure 3:**
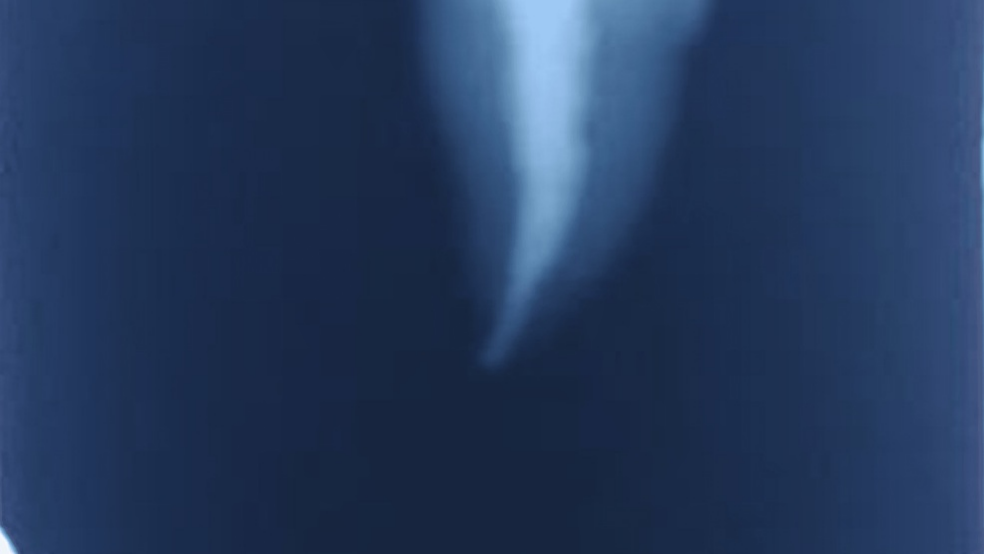
Postoperative radiograph.

In both groups (A and B), after condensing MTA at the apical third, the coronal portion was sealed with a cotton pellet and an intermediate restorative material (IRM). After two hours, the IRM and the cotton pellet were removed, and the canals were dried and obturated with gutta-percha (Dentsply Maillefer, Dentsply France SAS) using a lateral condensation method and zinc oxide eugenol sealer (Deepak Enterprise, Dental Products of India, India). Brothman has shown equivalent efficacy of vertical and lateral condensation in the obturation of permanent teeth [4]. Hence, lateral condensation was used in our study. Type IX Glass Ionomer (Fuji IX, GC Corporation, Japan) was used to seal the coronal portion of all samples. In group C, all samples (100) were used as control and no apical barrier was placed. The samples were obturated by lateral condensation method using gutta-percha and zinc oxide eugenol sealer and were verified by post-obturation radiographs in all three groups (Figure 3).

All samples were subjected to a thermocycling process in water between 5°C and 55°C for 100 cycles. The storage time in each bath was for 20 seconds. The transfer time between the baths was five seconds. All root segments were stored at 37°C in normal saline.

Transportation of samples

The extracted teeth were cleaned by ultrasonic scaling to remove soft tissue tags, debris, and calculus and later autoclaved. Initially, the samples were stored in 0.1% thymol for one month followed by normal saline at 37°C until further use. Infection control guidelines for dental care by the Centers for Disease Control and Prevention 2003 were followed. The teeth were transported in three well-constructed plastic containers with sealable lids, each containing 0.1% thymol solution. The containers were labeled with the group name and biohazard labels. The investigator and the assistant used protective eyewear, gloves, masks, head caps, and aprons. Waste samples were collected in red bags and disposed to the biomedical waste management team.

Measurement of fracture resistance (Instron testing machine)

**Figure 4:**
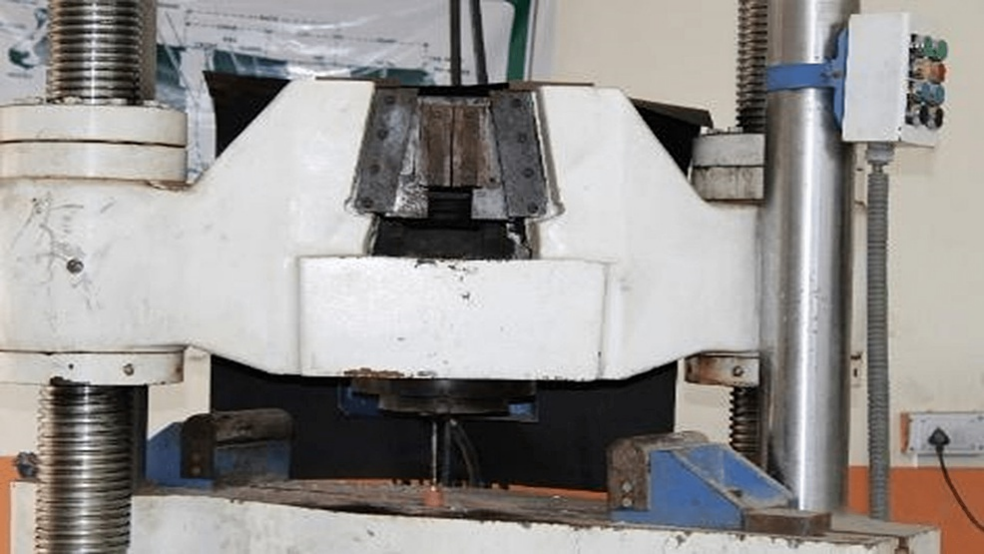
Instron Universal testing machine. 1000 Servo Computerized Instron Universal testing machine.

A prefabricated aluminum jig was used to fit each tooth individually. An Instron Universal Testing Machine (TUFC 1000 Servo Computerized Instron Universal Testing machine) was used to apply a specified load to each specimen at a crosshead speed of 5.0 mm/minute. Each specimen block was fixed in the aluminum jig, ensuring a loading angle of 90 degrees to the long axis of the tooth (Figure 4).

A total of 300 teeth were prepared for measuring the fracture resistance of group A (MTA Plus), group B (MTA Angelus), and group C (control). The load was delivered in a lingual-labile direction. The force was applied in a static manner and measured continuously using the Instron Universal testing machine until catastrophic failure, which resulted in a complete fracture of the cervical portion of the root. The peak load of fracture was measured in Newton (N) [5-7].

Statistical analysis

Data entry was done in Microsoft Excel spreadsheets, and descriptive and inferential statistical analysis was done using SYSTAT version 12 (Crane’s software, Bangalore, a licensed copy), and Graph Pad Insat 03 software. Statistical analysis was done using descriptive statistics such as mean, standard deviation, percentage, and proportions. All assessment variables under study were compared using Student’s unpaired t-test at a 5% (0.05) level of significance. P-values ≤0.05 were considered statistically significant (two-tailed). All groups were compared by applying the one-way analysis of variance (ANOVA) test (Tukey-Kramer multiple comparison test).

## Results

The mean fracture resistance values were 532.14 N, 540.81 N, and 460.63 N for group A (MTA Plus), group B (MTA Angelus), and group C (control), respectively (Table 1).

**Table 1:** Comparison of mean and SD values of fracture resistance of test materials (groups A and B) and the control group (group C). MTA = mineral trioxide aggregate; SD = standard deviation

Groups	Fracture resistance of test material (mean ± SD)
Group A (MTA Plus)	532.14 ± 5.19
Group B (MTA Angelus)	540.81 ± 3.56
Group C (control)	460.63 ± 7.91

By applying the one-way ANOVA test and post-hoc Tukey-Kramer multiple comparison test, there was a significant difference between the mean values of fracture resistance of test material (experimental groups A and B) with the control (group C) (p < 0.01) (Table 2).

**Table 2:** Results of fracture resistance after applying one-way ANOVA (Tukey’s multiple comparison test) for the three groups. DF = differentiation; ANOVA = analysis of variance

Source of variation	DF	Sum of squares	Mean square	Result
Treatments (between columns)	2	2,151.4	1,075.7	P = 0.0001, Significant
Residual (within columns)	79	2,045.7	25.895	
Total	81	4,197.1	41.541	

By applying the Student’s unpaired t-test for intergroup comparison, there was a statistically significant difference between the mean values of fracture resistance of test materials when group A (MTA Plus), group B (MTA Angelus), and group C (Unfilled group) was compared using Instron testing machine (p < 0.01). On intragroup comparison, MTA Angelus was better than MTA Plus and control (Table 3).

**Table 3:** Intergroup comparison of fracture resistance by applying Student’s unpaired t-test. MTA = mineral trioxide aggregate; SD = standard deviation; hs = highly significant

Intragroup comparison		Fracture resistance of the test materials (mean ± SD)		t-value	P-value
Group A (MTA Plus)	Group B (MTA Angelus)	532.14 ± 5.19 N	540.81 ± 3.56 N	12.607	0.001 (hs)
Group A (MTA Plus)	Group C (unfilled)	532.14 ± 5.19 N	460.63 ± 7.91 N	72.764	0.001 (hs)
Group B (MTA Angelus)	Group C (unfilled)	540.81 ± 3.56 N	460.63 ± 7.91 N	92.850	0.001 (hs)

## Discussion

MTA was introduced in endodontics by Mahmoud Torabinejad in the 1990s as a root-end filling material. For two decades, it has been one of the most desired biomaterials for research. The trioxide aggregate in MTA consists of aluminum, calcium, and selenium. MTA has several desirable properties, such as bioactivity, biocompatibility, hydrophilicity, radiopacity, sealing ability, and low solubility. One of the most important advantages of MTA in dentistry is its ability to set in a moist environment [7].

MTA was first introduced in 1993 and received FDA approval in 1998. In 1999, Pro Root MTA (Dentsply Tulsa Dental Specialties, Johnson City, TN) was the first commercially available MTA product launched in the United States. MTA Angelus (Angelus, Londrina, Brazil/Clinician’s Choice, New Milford, CT) was launched in Brazil in 2001 and received FDA approval in 2011, after which it was made available in the United States. MTA Plus (Prevest Denpro Limited, Jammu, India) was a finer powder, lower-cost product introduced in 2011 [8,9].

MTA Angelus is manufactured in small airtight containers, which makes it easy to store and reuse. MTA Angelus has a setting time of 15-45 minutes after preparation. MTA Angelus has a fast setting time due to less concentration of calcium sulfate, which is responsible for the delay in setting time in the original product [10]. The MTA Plus kit includes two mixing liquids, namely, a proprietary salt-free polymer gel and water. Different setting times and physical-rheological properties can be obtained using the gel and varying the powder-to-gel ratio [11]. The gel was formulated to confer washout resistance, whereas its fine powder particle size improved the handling and placement. The setting time of MTA Plus is 20-40 minutes [12].

Andreasen et al. conducted a study in 2006 comparing the fracture resistance of calcium hydroxide and MTA as a root-filling material after various treatment modalities. The incisors, with approximately 80% root formation, were extracted from the jaws of slaughtered sheep and divided into four experimental groups. The study showed that MTA increased the fracture resistance of teeth in comparison with calcium hydroxide, and hence, is a better root-filling material compared to calcium hydroxide [13]. In our study, we evaluated the fracture resistance of two commercially available MTAs, namely, MTA Angelus and MTA Plus. Our results showed that the teeth without any filling (group C, control group = 460.63 ± 7.91 N) showed the lowest fracture resistance in comparison to the MTA Angelus (group B = 540.81 ± 3.56 N) and MTA Plus (group A = 532.14 ± 5.19 N). Both the tested materials considerably strengthened the immature teeth. Statistically significant differences were observed between the two materials, which may be attributed to the composition and structure of MTA Angelus and MTA Plus, which was in conjunction with the study by Tuna et al. [10] who assessed the long-term fracture resistance of human immature permanent teeth filled with Bio Aggregate (BA), MTA and calcium hydroxide. Considering the long-term risk of cervical root fracture associated with immature teeth, the use of DiaRoot-BA as a root canal filling material appeared to be the most advantageous of the materials tested.

MTA Plus contains anhydrite and ettringite, which are responsible for additional strength, thereby increasing the fracture resistance, but it was found to be lower when compared with MTA Angelus. Other studies by Guven et al. have shown MTA Plus to have greater strength than MTA Angelus [13]. The results of our study showed different results from the study by Guven et al. Our study showed that the presence of ettringite and anhydride in MTA Plus did not increase the strength.

Turker et al. assessed the fracture resistance of teeth with simulated perforating internal resorption cavities repaired with different calcium silicate-based cement and backfilling materials such as MTA, MTA Plus, and Biodentine [11]. The fracture-resistant property of MTA was also evaluated by Chunn et al. who compared MTA with Biodentine as a root-filling material using the finite element method and concluded that MTA is better than Biodentine [14].

In future studies, along with the fracture resistance, other parameters such as shear strength, push-out bond strength, apical sealing ability, and molecular analysis can be assessed, as well as a comparison of MTAs with other novel bioceramics such as Biodentine, EndoSequence, and calcium-enriched mixtures should also be considered [15]. While the limitations of this study were that more parameters such as shear strength and wash-out resistance were not considered. Consideration of these parameters would give more clear knowledge about the materials and would help in choosing a better material for treatment.

## Conclusions

Root end filling is a crucial step in endodontic treatment to avoid the fracture of immature teeth. Various root end-filling materials such as calcium hydroxide, MTA Plus, MTA Angelus, and Biodentine have been assessed for fracture resistance. According to the findings of this study, the composition and structural changes of MTA Angelus (group B) make it more fracture resistant than MTA Plus (group A). The teeth without any filling (control group) showed the lowest fracture resistance. Hence, root filling material is mandatory for providing strength to immature teeth while performing endodontic procedures.
